# Sexual orientation and gender identity differences in perceptions and product appeal in response to e-cigarette advertising

**DOI:** 10.18332/tid/169739

**Published:** 2023-09-01

**Authors:** Jessica Liu, Joanne G. Patterson, Brittney Keller-Hamilton, Donghee N. Lee, Kirsten R. Chrzan, Elise M. Stevens

**Affiliations:** 1 REACH Lab, Division of Adolescent Medicine, Department of Pediatrics, Stanford University, Stanford, United States; 2 Division of Epidemiology, The Ohio State University College of Public Health, The Ohio State University, Columbus, United States; 3 The Ohio State University Comprehensive Cancer Center, The Ohio State University, Columbus, United States; 4 Department of Internal Medicine, The Ohio State University College of Medicine, The Ohio State University, Columbus, United States; 5 Center for Tobacco Research, The Ohio State University Comprehensive Cancer Center, The Ohio State University, Columbus, United States; 6 Division of Preventive and Behavioral Medicine, Department of Population and Quantitative Health Sciences, University of Massachusetts Chan Medical School, Worcester, United States

**Keywords:** e-cigarette, advertising, sexual minority, gender

## Abstract

**INTRODUCTION:**

E-cigarette use is disparately high among sexual minoritized populations. As e-cigarette advertising may influence product appeal, this study tested sexual orientation- and gender-based differences in response to e-cigarette advertisement exposure on advertisement perceptions and product appeal.

**METHODS:**

We recruited 497 adults (mean age=31.9 years, 45.1% women, 54.3% heterosexual, 71.2% Non-Hispanic White) living in the United States via the crowdsourcing platform Prolific. Participants viewed two randomly selected e-cigarette advertisements (from n=173 advertisements). Post-exposure, participants rated the perceived advertisement effectiveness, relevance, and product use intention. Associations between sexual orientation and outcomes were estimated using multivariable linear mixed-effects models. We tested interaction effects between sexual orientation, gender, and advertisement feature (e.g. presence of humans, flavors, and product packaging), and ran Tukey post hoc tests for pairwise comparisons.

**RESULTS:**

Post-exposure, heterosexual women, sexual minoritized men, and sexual minoritized women (reference group: heterosexual men) rated perceived advertisement effectiveness and relevance lower after viewing advertisements featuring flavors (vs no flavors; all p<0.001). Sexual minoritized men and sexual minoritized women rated perceived advertisement relevance lower after viewing advertisements featuring humans (all p<0.001) or fruit (all p<0.001). Heterosexual women, sexual minoritized men, and sexual minoritized women reported lower product use intention after viewing advertisements featuring an e-liquid bottle (vs no e-liquid bottle; all p<0.05).

**CONCLUSIONS:**

Sexual minoritized women and men reported lower e-cigarette advertisement appeal and product use intentions than heterosexual men. More evidence is needed to understand advertisement perceptions and product appeal in this group to inform e-cigarette advertising regulations and anti-tobacco messaging campaigns that aim to reduce tobacco-related health inequities.

## INTRODUCTION

E-cigarette use is disparately high among sexual minoritized populations (i.e. lesbian, gay, bisexual, or other non-heterosexual people), with 13.2% of US adults within this population reporting current use, over three times that of heterosexual adults (4.1%)^1^. However, these differences vary across subpopulations of sexual minoritized adults. For example, research using the 2016–2018 Population Assessment of Tobacco and Health (PATH) Study data found that 21.7% of bisexual men aged 18–29 years and 13.2% of bisexual men aged 30–44 years reported past 30-day e-cigarette use (vs 15.7% and 8.8% of heterosexual men, respectively)^2^. However, past 30-day e-cigarette use rates were lowest among gay men aged 18–29 years (12.9%) and 30–44 years (7.5%)^2^. Among women, past 30-day e-cigarette use rates were lowest among heterosexual women aged 18–29 years (7.9%) and 30–44 years (5.0%), compared to lesbian (17.6%) and bisexual (20.9%) women aged 18–29 years, and lesbian (16.4%) and bisexual (19.0%) women aged 30–44 years^2^. In general, tobacco product use tends to be higher among sexual minoritized women, particularly those who identify as bisexual^2^.

The factors underlying e-cigarette use disparities among sexual minoritized populations are multilevel and complex. Generally, sexual minoritized adults describe tobacco use as a strategy to resist and cope with excess stress arising from structural violence and minority stress (i.e. excess stress arising from discrimination based on one’s minoritized sexual orientation or gender identity)^3,4^. Tobacco use is also described as a ‘counterculture’ strategy to resist social norms^5^, a means to build social connections within lesbian, gay, bisexual, and transgender (LGBT) communities^6^, and a way to signal one’s LGBT identity and belonging^6^. Big Tobacco’s historically targeted marketing to LGBT groups (e.g. Project Subculture Urban Marketing in San Francisco, California) is likely a contributing factor to marked e-cigarette use disparities among sexual minoritized people^7^. The tobacco industry has consistently tailored advertisements to LGBT populations while also placing targeted marketing in LGBT publications and event spaces^8^. Yet, until recently, tobacco prevention and control efforts have largely overlooked sexual minoritized populations, including the role of tobacco marketing in e-cigarette product appeal and use.

Emerging literature has found that compared to their heterosexual peers, sexual minoritized adults have increased exposure to tobacco advertising and find tobacco industry marketing more appealing^9^. However, there are differences by sexual orientation and gender. For example, in a study of US adults, sexual minoritized adults (both men and women) generally reported more exposure to tobacco industry marketing than their heterosexual peers, yet only lesbian and bisexual women were more receptive to this marketing (vs heterosexual women)^9^. In contrast, in a nationally representative sample of young adults in the US, bisexual women reported greater exposure to e-cigarette marketing than heterosexual women, but these differences were not evident for young adult lesbian women or gay and bisexual men (vs heterosexual women and men, respectively)^10^. These disparities in tobacco advertising exposure among sexual minoritized groups are further exacerbated by the intersectionality of race, ethnicity, and gender^10,11^, as the tobacco industry also has a history of targeting these groups^11^. For example, in a study of US young adults, bisexual Hispanic women had higher exposure to e-cigarette, cigarette, and cigar advertisements (vs non-Hispanic White heterosexual women), whereas Black bisexual women had higher exposure to cigarette and cigar marketing (vs non-Hispanic White heterosexual women) and Black bisexual men had higher exposure to cigar advertisements (vs non-Hispanic White heterosexual men). However, there were no differences by race or ethnicity among lesbian women or gay men^10^. These findings underscore the importance of examining differences in tobacco marketing exposure among gender-based subgroups of sexual minoritized adults.

Excess exposure to tobacco industry marketing among the sexual minoritized populations is concerning, as prior evidence links e-cigarette advertisement exposure to product appeal and use^12^. A study found that sexual minoritized adults were more likely to have viewed and interacted with (i.e. searched for and/or shared) tobacco advertisements, compared to their heterosexual peers^13^. This study also found that sexual minoritized participants reported significantly higher odds of past 30-day e-cigarette and tobacco use^13^.

However, little is known about how exposure to e-cigarette advertising is associated with product appeal among sexual minoritized (vs heterosexual) populations. This is a critical gap, as the US Food and Drug Administration (US-FDA) needs scientific evidence to regulate e-cigarette advertisements that may exacerbate disparities. Research on the appeal of specific e-cigarette advertisement features has mainly focused on the general population of youth and young adults. Such research has found that certain features, such as the presence of humans, flora imagery, and natural descriptors, are associated with increased appeal in this younger age category^14,15^. An eye-tracking study found gender-based differences in time of viewing certain advertisements^14^, which may also suggest that there are differences in the impact of advertisement features based on gender and sexual identity. Thus, understanding how the perceived effectiveness and relevance of certain e-cigarette advertisement features appeal to other subgroups, such as sexual minoritized people, may help reduce e-cigarette and tobacco use disparities.

Our main research aim was to identify whether certain popular e-cigarette advertisement features were more appealing to sexual minoritized people. Within e-cigarette advertisements, key features might be more appealing than others in specific populations. Thus, the current study tested the effect of exposure to e-cigarette advertisements with key features on advertisement perceptions and product appeal among heterosexual and sexual minoritized women and men. We hypothesized that e-cigarette advertisements would be more appealing to sexual minoritized women and men, compared to heterosexual women and men.

## METHODS

### Sample

We conducted an online experiment among 497 participants. Participants were enrolled through the online consumer research panel Prolific in October 2021. Participants were eligible to enroll in the study if they were aged ≥18 years and resided in the US. We oversampled sexual minoritized participants to have enough power to answer our research question^16^. In this study, we used Prolific’s screening tools to determine the eligibility of participants, as Prolific only allows participants to opt-in to the study if they meet the specific requirements of the study. We aimed to recruit a sample of equal number heterosexual individuals (n=250) and sexual minoritized individuals (n=250) (i.e. reported sexual orientation as homosexual, bisexual, asexual, or other in Prolific’s prescreening). Once participants met the eligibility criteria, they were able to opt-in to the study. Once quotas for each group were met, the study was not available to members of that specific group.

### Study design

Consented participants began the online survey by reporting their e-cigarette, tobacco, and alcohol use. They then viewed two e-cigarette advertisements, randomly selected from a sample of 173 advertisements that were identified as including popular features (e.g. humans in the advertisements, flavors, and product packaging), with an equivalent number of advertisements representative of each feature^17^. Advertisements were selected from a previous content analysis of e-cigarette advertisement features^17^. Advertisements with features that appeared in at least 5% of the ads were chosen to further explore in this study^15^. This methodology was based on previous work done to examine cigarette advertising^15^. Multiple advertisements with the same feature were used in the study to account for one-message design error, which ensured that message responsiveness was attributed to the particular feature and not to any other aspect of a singular message (e.g. color, design, layout)^18^. Advertisements came from media, including email, print magazines and mail, social media sites, and e-cigarette company websites^17^. Participants were allowed to view the advertisements for as long as they wanted. Participants then completed post-assessment measures, including perceptions of the advertisement and product use intentions. After completion, participants were compensated $3.96 according to Prolific’s policies.

### Measures

*Demographics*

Participants reported their age, race/ethnicity (Non-Hispanic White, Non-Hispanic Black, Other/Multiple, Hispanic), and individual income ($) (<50000 or ≥50000)^16^.

*Sexual orientation and gender identity*

First, participants were asked: ‘What is your gender?’ with possible answer options of ‘Female’, ‘Male’, ‘Trans female/Trans woman’, ‘Trans male/Trans man’, ‘Genderqueer/Gender non-conforming/Gender expansive’, and ‘Other’. For sexual orientation, participants were asked: ‘Which of the following best describes you?’ with possible answer options of ‘Straight or heterosexual’, ‘Lesbian or Gay’, ‘Bisexual’, and ‘Other non-heterosexual identity’.

With respect to sexual orientation, ‘Lesbian and Gay’ (n=57), ‘Bisexual’ (n=142), and ‘Other non-heterosexual orientation’ (n=28) were combined and recoded as ‘Sexual minoritized’ to prevent small cell sizes at the intersection of sexual orientation and gender identity (SOGI) for our multivariable analyses. We then created a multi-categorical SOGI variable as follows: heterosexual man, heterosexual woman, sexual minoritized man, sexual minoritized woman. Our final analytic sample for multivariable analyses comprised 462 participants.

*Perceived advertisement effectiveness*

Perceived advertisement effectiveness was assessed using a five-question instrument that asked whether participants thought the advertisement was memorable, attention grabbing, powerful, convincing, and meaningful^19^. Scores on a 5-point Likert scale ranged from 1 (strongly disagree) to 5 (strongly agree). Scores were averaged across the five questions (Cronbach’s alphas >0.93).

*Perceived advertisement relevance*

Perceived advertisement relevance was assessed using two questions: ‘The ad seemed to be written personally for me’, and ‘The ad was very relevant to my situation’^20^. Scores from a 5-point Likert scale ranged from 1 (strongly disagree) to 5 (strongly agree)^16^. Scores were averaged across the two questions (Cronbach’s alphas >0.90)^16^.

*Product use intention*

Product use intention was assessed using a single item: ‘This ad made me want to use the product’ on a 5-point Likert scale of 1 (strongly disagree) to 5 (strongly agree)^21^.

*E-cigarette advertisement features*

Advertisements were taken from four of the most popular e-cigarette brands (Blu, JUUL, Puff Bar, and Vuse) between 2019 and 2020^22^. Complete information on the advertisements and procedures of the content analysis can be found elsewhere^17^. Features that appeared in at least 5% of the advertisements were tested in the current study^15^, including: the presence of non-tobacco flavor; the presence of a human(s); price reduction; product packaging; e-liquid bottle; actual product; product in use; ‘satisfying’ descriptor; claim that the product was an alternative to or exchangeable with a cigarette; fruit imagery (e.g. apples, cherries); flora imagery (e.g. plants, leaves); positive sensation appeal (e.g. good taste, smell); and camaraderie or friendship appeal (e.g. closeness with others)^17^.

### Statistical analysis

Using descriptive statistics, we report distributions of all variables stratified by SOGI, including the outcome measures: perceived advertisement relevance, perceived advertisement effectiveness, and product use intention^16^. As the evidence indicates a marked heterogeneity in e-cigarette use among US adults by SOGI^11^, we assessed differences in outcomes at the intersection of these identities. We used linear mixed-effects models, fit with restricted maximum likelihood estimation, to assess interactions between the sexual orientation (heterosexual/sexual minoritized) and gender identity (man/woman) variables with each outcome variable. All models included random intercepts for each participant and fixed effects for age, race/ethnicity, and income. We conducted post-estimation pairwise comparisons, adjusting the alpha using the Tukey test. We used heterosexual men as our reference group, as research shows that the association between sexual identity and tobacco use is stronger for women^11^.

To assess the influence of advertisement features, we used linear mixed-effects models, fit with restricted maximum likelihood estimation, to estimate associations of the SOGI variable and advertisement feature with each outcome variable. All models included random intercepts for each participant and fixed effects for age, race/ethnicity, and income. Finally, we ran adjusted linear mixed-effects models that included product interaction terms between the SOGI variable and each feature (in separate models) and assessed their statistical significance using partial F-tests. Statistical significance of fixed effects, including the interaction terms, was assessed using a partial F-test with an alpha of 0.05 (two-tailed). In the models where the interaction between the SOGI variable and advertisement feature was statistically significant, we report stratified results and conduct post-estimation pairwise comparisons, adjusting the alpha using the Tukey test. We used R software [version 1.1.456] for all analyses.

With respect to gender, few respondents identified as non-binary (n=25) or transgender (n=10). While data from transgender and non-binary (TNB) participants was too limited for multivariable statistical analyses at the intersection of gender and sexual orientation, we included descriptive statistics (Table 1), as these data can be applied in future meta-analyses^23^. For multivariable analyses, TNB participants were excluded, and gender was recoded as ‘woman’ and ‘man’.

**Table 1:** Participant demographics of 2021 brief online advertisement exposure study (N=497)

	*Overall (N=497) n (%)*	*Heterosexual men (N=188) n (%)*	*Heterosexual women (N=82) n (%)*	*Sexual minoritized men (N=50) n (%)*	*Sexual minoritized women (N=142) n (%)*	*Transgender/Non-binary^b^ (N=35) n (%)*	*p^a^*
**Age** (years), mean ± SD	31.9 ± 10.6	31.7 ± 11.1	36.9 ± 9.6	26.8 ± 8.9	32.5 ± 10.2	26.1 ± 8.3	**<0.001**
**Gender**							
Woman	224 (45.1)						
Man	238 (47.9)						
Transgender/Non-binary	35 (7.0)						
**Sexual orientation**							
Straight or heterosexual	270 (54.3)						
Sexual minoritized	227 (45.7)						
**Race/ethnicity**							**0.003**
Non-Hispanic White	352 (71.2)	133 (70.7)	62 (75.6)	33 (66.0)	96 (67.6)	29 (82.9)	
Non-Hispanic Black	56 (11.3)	31 (16.5)	6 (7.3)	6 (12.0)	13 (9.2)	0 (0.0)	
Hispanic	33 (6.7)	10 (5.3)	5 (6.1)	4 (8.0)	10 (7.0)	4 (11.4)	
Other/Multiple	54 (10.9)	14 (7.4)	9 (11.0)	7 (14.0)	23 (16.2)	1 (2.9)	
**Income** ($)							**<0.001**
<50000	270 (54.3)	50 (26.6)	57 (69.5)	25 (50.0)	109 (76.8)	29 (82.9)	
≥50000	227 (45.7)	138 (73.4)	25 (30.5)	25 (50.0)	33 (23.2)	6 (17.1)	
**E-cigarette use^c^**							**<0.001**
Never	167 (33.6)	53 (28.2)	35 (42.7)	21 (42.0)	49 (34.5)	9 (25.7)	
Ever	122 (24.5)	26 (13.8)	30 (36.6)	10 (20.0)	40 (28.2)	16 (45.7)	
Past 30-day	208 (41.9)	109 (58.0)	17 (20.7)	19 (38.0)	53 (37.3)	10 (28.6)	
**Cigarette use**							**<0.001**
Never	158 (31.8)	35 (18.6)	31 (37.9)	12 (24.0)	68 (47.9)	12 (34.3)	
Ever	166 (33.4)	44 (31.0)	31 (37.9)	24 (48.0)	44 (31.0)	19 (54.3)	
Past 30-day	173 (34.9)	30 (21.1)	20 (24.4)	14 (28.0)	30 (21.1)	4 (11.4)	
**Alcohol use**							**<0.001**
Never	24 (4.8)	8 (4.3)	4 (0.5)	3 (6.0)	8 (5.6)	1 (2.9)	
Ever	74 (14.9)	12 (6.4)	14 (17.1)	8 (16.0)	27 (19.0)	13 (37.1)	
Past 30-day	399 (80.3)	168 (89.4)	64 (78.0)	39 (78.0)	107 (75.4)	21 (60.2)	
**Perceptions and intention**							
Perceived advertisement effectiveness score (1–5), mean ± SD	2.6 ± 1.2	3.2 ± 1.2	2.2 ± 1.1	2.0 ± 1.1	2.2 ± 1.0	2.3 ± 1.1	**<0.001**
Perceived advertisement relevance score (1–5), mean ± SD	2.1 ± 1.3	2.9 ± 1.4	1.6 ± 0.9	1.5 ± 0.9	1.6 ± 0.9	1.8 ± 1.1	**<0.001**
Product use intention score (1–5), mean ± SD	2.3 ± 1.4	3.1 ± 1.5	1.7 ± 1.0	1.6 ± 1.0	1.8 ± 1.1	2.0 ± 1.2	**<0.001**

a The p-values were assessed through Pearson’s chi-squared test. TNB participants were not included in the chi-squared tests to be consistent with our multivariable statistical analyses.

b Descriptive data were included for transgender and non-binary participants. As the sample size was too small for meaningful analysis by gender and sexual orientation, this group was excluded from multivariable statistical analyses.

c Participants were asked to report their e-cigarette use and if they had ever ‘used an electronic cigarette (e-cigarette), even one or two times?’. If participants answered ‘Yes’, they were then asked, ‘During the past 30 days, on how many days did you use an e-cigarette?’. E-cigarette use status categorized as ‘current use’ if they used an e-cigarette in the past 30 days, ‘ever use’ if they ever used e-cigarettes but reported 0 days of e-cigarette use in the past 30 days, and ‘never use’ if they responded ‘No’ to ever using an e-cigarette even one or two times16. Participants also reported their use of combustible cigarettes and alcohol. We recoded participants to ‘current’, ‘ever’, and ‘never’ users of combustible cigarettes and alcohol similar to how we categorized e-cigarette use status.

## RESULTS

### Participants

Our sample (Table 1) included 497 participants with a mean age of 31.9 years (SD=10.6). Approximately half of the participants identified as women (45.1%) and men (47.9%), and 7% identified as transgender/non-binary. The majority of participants identified as straight or heterosexual (54.3%), Non-Hispanic White (71.2%), and had an income ($) <50000 (54.3%)^16^. Of the participants who identified as men in our study (n=238), 21% identified as sexual minoritized men, and 63.4% of those who identified as women in our study (n=224) identified as sexual minorized women.

### Product appeal and use intentions by sexual orientation and gender identity

Perceived advertisement effectiveness score (Table 2) was lower for heterosexual women (mean=2.2, SD=1.1, p=0.001), sexual minoritized women (mean=2.2, SD=1.0, p<0.001), and sexual minoritized men (mean=2.0, SD=1.1, p<0.001) than heterosexual men (mean=3.2, SD=1.2).

**Table 2:** Mean scores and pairwise differences for perceived advertisement effectiveness, advertisement relevance, and product use intention of 2021 brief online advertisement exposure study (N=462)

	*Heterosexual men Mean (SD)*	*Heterosexual women Mean (SD)*	*Sexual minoritized men Mean (SD)*	*Sexual minoritized women Mean (SD)*
Perceived advertisement effectiveness score (1–5)	3.2 (1.2)^a,b,c^	2.2 (1.1)^a^	2.0 (1.1)^b^	2.2 (1.0)^c^
Perceived advertisement relevance score (1–5)	2.9 (1.4)^a,b,c^	1.6 (0.9)^a^	1.5 (0.9)^b^	1.6 (0.9)^c^
Product use intention score (1–5)	3.1 (1.5)^a,b,c^	1.7 (1.0)^a^	1.6 (1.0)^b^	1.8 (1.1)^c^

Pairwise differences between groups (p<0.05) from post hoc tests from the linear mixed-effects models are noted with a shared subscript in each row.

Advertisement relevance score (Table 2) was lower for heterosexual women (mean=1.6, SD=0.9, p<0.001), sexual minoritized women (mean=1.6, SD=0.9, p<0.001), and sexual minoritized men (mean=1.5, SD=0.9, p<0.001) than heterosexual men (mean=2.9, SD=1.4).

With respect to product appeal score (Table 2) , heterosexual women (mean=1.7, SD=1.0, p<0.001), sexual minoritized women (mean=1.8, SD=1.1, p<0.001), and sexual minoritized men (mean=1.6, SD=1.0, p<0.001) reported lower intentions to use e-cigarettes than heterosexual men (mean=3.1, SD=1.5).

No outcome differences were found, between heterosexual and sexual minoritized women, or sexual minoritized women and men.

### Associations between gender, sexual orientation, feature, and perceptions of advertisements and e-cigarettes

*Perceived advertisement effectiveness*

SOGI modified the effect of flavors (p=0.004), humans present (p=0.045), and product packaging shown (p=0.045) on perceived advertisement effectiveness. Among heterosexual women, sexual minoritized men, and sexual minoritized women, perceived advertisement effectiveness was rated lower for advertisements with flavors compared to advertisements without flavors (all p<0.001; Supplementary file Table 1). Among sexual minoritized men and sexual minoritized women, perceived advertisement effectiveness was rated lower for advertisements with humans present compared to advertisements without humans (all p<0.001; Supplementary file Table 1). Among heterosexual women, sexual minoritized men, and sexual minoritized women, perceived advertisement effectiveness was rated lower for advertisements with product packaging shown compared to advertisements without product packaging (all p<0.001; Supplementary file Table 1).

*Perceived advertisement relevance*

SOGI modified the effect of flavors (p=0.031), humans present (p=0.014), and fruit imagery (p=0.039) on perceived advertisement relevance. Among heterosexual women, sexual minoritized men, and sexual minoritized women, advertisements with flavors were rated lower in perceived advertisement relevance than those without that feature (all p<0.001; Supplementary file Table 1). Among heterosexual women, sexual minoritized men, and sexual minoritized women, perceived advertisement relevance was rated lower for advertisements with humans compared to those without humans (all p<0.005; Supplementary file Table 1). Among heterosexual men, heterosexual women, sexual minoritized men, and sexual minoritized women, advertisements with fruit imagery were rated as less relevant than advertisements without that feature (all p<0.03; Supplementary file Table 1).

*Product use intention*

SOGI modified the effect of e-liquid bottle shown (p=0.046) and ‘Alternative to Cigarettes’ claim (p=0.026) on product use intention. Among heterosexual women, sexual minoritized men, and sexual minoritized women, product use intention was rated lower for advertisements with an e-liquid bottle shown compared to advertisements without that feature (all p<0.05; Supplementary file Table 1). Among sexual minoritized women, product use intention was rated lower for advertisements with an ‘Alternative to Cigarettes’ claim compared to advertisements without that feature (all p<0.05; Supplementary file Table 1).

## DISCUSSION

Contrary to our hypotheses, sexual minoritized women and men did not report greater perceived advertisement effectiveness or perceived advertisement relevance after viewing real-world e-cigarette advertisements, nor did they report higher intentions to use e-cigarettes. With respect to advertisement features, SOGI modified the effect of flavors and humans on both advertisement effectiveness and advertisement relevance. SOGI also modified the effect of featuring e-liquid bottles and ‘Alternative to Cigarettes’ claims in advertisements on product use intention. Overall, we found that sexual minoritized participants tended to find such features less appealing and had lower product use intentions after viewing these advertisements, compared to heterosexual men.

In the general population, exposure to e-cigarette advertisements is associated with subsequent e-cigarette use across all age groups (youth and adults)^24,25^. Some evidence suggests that exposure to tobacco marketing increases the risk of cigarette smoking among sexual minoritized adults^26^. It is possible that, given the history of targeted marketing directed toward sexual minoritized communities and increased public skepticism toward ‘rainbow washing’^27^ (that is, culturally-targeted marketing leveraging rainbow colors or imagery to indicate support and increase credibility with sexual minoritized communities) that sexual minoritized adults in our study have developed a level of media literacy and accompanying skepticism toward tobacco industry marketing, including e-cigarette advertisements. Qualitative studies have described how sexual and gender minoritized participants have felt targeted by the tobacco industry and, thus, were skeptical towards pro- and anti-tobacco messages^8^. Survey data additionally suggest that advertising skepticism may be a protective factor among sexual minoritized adults for tobacco use^28^. Given these findings and our results, additional population-level studies are needed to understand why sexual minoritized adults use e-cigarettes more than other groups^29^, and if advertising skepticism is protective of e-cigarette use among sexual minoritized adults despite their increased exposure to targeted tobacco advertising.

We found substantial heterogeneity in responses to e-cigarette advertisement features such that, unlike heterosexual men, sexual minoritized women and men found advertisements featuring flavors, fruit imagery, e-cigarette products, or humans, less relevant than advertisements that did not include those features. These findings contrast with findings by Chen-Sankey et al.^30^, in which exposure to advertisements with flavors and people was positively associated with e-cigarette product appeal among mostly heterosexual young adults. Similar findings have also been shown for cigarette advertisements and product appeal with heterosexual adolescents and young adults^15^. The contrasting findings may have been due to the fact that participants in our study were only briefly exposed to two advertisements in an online study rather than having a longer exposure period in the real world. Given that these features are commonly used in e-cigarette advertising^17^, our findings suggest that regulating these features may reduce use in the general population while failing to attenuate e-cigarette use disparities.

It is important to note that this study included advertisements from the most popular e-cigarette brands, which may not have been marketing to specific subgroups but aimed at the general population. In the US general population, uptake and use of e-cigarettes has been highest among men^31^, and more men receive e-cigarette promotions by direct mail marketing^32^. Prior studies have identified gendered-appeals in online e-cigarette marketing targeting men, including the portrayal of e-cigarette use as more attractive to romantic/sexual partners and as a way to increase social status^33^. Consequently, our findings that message effectiveness and product appeal was highest among heterosexual men may reflect the use of marketing tactics that generally target men as the primary consumers of e-cigarettes.

Given the tobacco industry’s history of predatory marketing directed to sexual minoritized populations, it is possible that culturally specific advertisement features (i.e. those leveraging imagery and messaging that emphasize the cultural values and social identities of sexual minoritized communities) may differently impact advertisement perceptions, product appeal, and use among this population. Future studies comparing the effect of culturally targeted e-cigarette advertisements versus general e-cigarette advertisements may illuminate advertisement features that unduly influence product appeal among sexual minoritized populations and, thus, may be targeted in regulatory efforts to reduce health inequities.

### Limitations

Although our study explored a novel topic regarding the appeal of e-cigarette advertisements to sexual minoritized groups, it has the following limitations. We had to exclude non-binary and transgender participants from multivariable analyses due to small cell size; however, descriptives for this group are included in Supplementary file Table 2. Additionally, all gender minoritized participants in our sample also reported a minoritized sexual orientation, but sample size precluded descriptive statistics at the intersection of gender identity and sexual orientation. Given increased nicotine and tobacco use among gender minoritized populations, future research must recruit larger samples of non-binary and transgender people. There may be additional heterogeneity in responses to advertisement features according to age group or e-cigarette/tobacco use status within gender and sexual minoritized groups (e.g. bisexual vs lesbian women)^16^, but we could not assess these multiple intersections due to sample size. Descriptive statistics for these groups are included in Supplementary file Table 2. Additionally, the findings of this study may not represent those who do not use the recruitment platform Prolific, and may have an overrepresentation of White participants (71.2%), and a lack of information from Asian and Pacific Islander groups. Thus, our sample population may not be generalizable to a national level. Yet, studies have found Prolific data to be of high quality^34^ and have shown to mirror the demographics of the US national population^35^.

Our measure of product use intention was through self-report, not behavioral outcomes. However, research has shown that intentions are a strong predictor of actual future health behaviors^36^. We also used real world advertisements, which can present issues due to the inability to manipulate specific elements of the advertisement. We did not include any validity checks to ensure that participants noticed the features being tested and did not include an equivalent number of advertisements representative for each feature. Yet, utilizing real world advertisements provides ecological validity, and we showed each participant multiple advertisements to reduce message design error. In other words, the design of the study is such that message responsiveness, on average, is likely attributed to the feature of the advertisement and not to any other aspect of the message (e.g. color, design, layout)^18^. We did not examine whether type of advertisement was associated with feature and study outcomes in our models.

## CONCLUSIONS

Our study identified substantial heterogeneity across sexual minoritized groups in appeal of various e-cigarette advertisement features. Although we found that sexual minoritized groups tended to rate certain features – including the presence of flavors, humans, and e-liquid bottles – as less appealing than do heterosexual men, this points to the need for additional research to identify which advertisement features sexual minoritized groups do find appealing, and which should be regulated to reduce disparities. Moreover, future studies should investigate longitudinal associations between exposure to advertising and subsequent e-cigarette use among sexual minoritized populations. Researchers, clinicians, and policymakers must continue to prioritize sexual minoritized groups when considering tobacco cessation, intervention work, and policy implementation, to help reduce tobacco use disparities.

## Data Availability

The data supporting this research are available from the authors on reasonable request.
