# Supplementary material for: Sexual orientation and gender identity differences in perceptions and product appeal in response to e-cigarette advertising
Source: Tob Induc Dis. 2023 Sep 1;21:111. doi: 10.18332/tid/169739 (PMC10472342; doi:10.18332/tid/169739)
Supplement: Supplementary file 1 [file TID-21-111-s1.pdf]

Supplemental Table 1: Adjusted associations between sexual orientation and gender identity, feature, their interaction, and outcomes (perceived advertisement effectiveness, perceived advertisement relevance, product use intention) of 2021 Brief Online Advertisement Exposure Study (N=462)

	Beta	95% Confidence Interval (lower limit)	95% Confidence Interval (upper limit)	p-value	Overall p-value
**Perceived Advertisement Effectiveness†**					
Flavors					**0.004**
Heterosexual men					
No flavors	Ref				
Flavors	0.26	-0.01	0.53	0.064	
Heterosexual women					
No flavors	Ref				
Flavors	-1.00[a]	-1.31	-0.69	**<0.001**	
Sexual Minoritized men					
No flavors	Ref				
Flavors	-1.06[a]	-1.41	-0.71	**<0.001**	
Sexual Minoritized women					
No flavors	Ref				
Flavors	-0.94[a]	-1.21	-0.67	**<0.001**	
Humans					**0.045**
Heterosexual men					
No humans	Ref				
Humans	-0.13	-0.35	0.09	0.248	
Heterosexual women					
No humans	Ref				
Humans	-0.30	-0.71	0.11	0.153	
Sexual Minoritized men					
No humans	Ref				
Humans	-1.16[a]	-1.65	-0.67	**<0.001**	
Sexual Minoritized women					
No humans	Ref				
Humans	-0.59[a]	-0.94	-0.24	**0.001**	
Product Packaging Shown					**0.045**
Heterosexual men					
No product packaging	Ref				
Product packaging shown	0.21	-0.01	0.43	0.055	
Heterosexual women					
No product packaging	Ref				
Product packaging shown	-0.85[a]	-1.18	-0.52	**<0.001**	
Sexual Minoritized men					
No product packaging	Ref				
Product packaging shown	-0.93[a]	-1.34	-0.52	**<0.001**	
Sexual Minoritized women					
No product packaging	Ref				

Product packaging shown	-0.95[a]	-1.24	-0.66	**<0.001**	
**Perceived Advertisement Relevance†**					
Flavors					**0.031**
Heterosexual men					
No flavors	Ref				
Flavors	0.07	-0.15	0.29	0.538	
Heterosexual women					
No flavors	Ref				
Flavors	-1.10[a]	-1.41	-0.79	**<0.001**	
Sexual Minoritized men					
No flavors	Ref				
Flavors	-1.05[a]	-1.40	-0.70	**<0.001**	
Sexual Minoritized women					
No flavors	Ref				
Flavors	-0.85[a]	-1.14	-0.56	**<0.001**	
Humans					**0.014**
Heterosexual men					
No humans	Ref				
Humans	-0.13	-0.31	0.05	0.154	
Heterosexual women					
No humans	Ref				
Humans	-0.56	-0.95	-0.17	**0.005**	
Sexual Minoritized men					
No humans	Ref				
Humans	-1.27[a]	-1.72	-0.82	**<0.001**	
Sexual Minoritized women					
No humans	Ref				
Humans	-0.76[a]	-1.09	-0.43	**<0.001**	
Fruit					**0.039**
Heterosexual men					
No Fruit	Ref				
Fruit	-0.23	-0.43	-0.03	**0.029**	
Heterosexual women					
No Fruit	Ref				
Fruit	-1.07[a]	-1.48	-0.66	**<0.001**	
Sexual Minoritized men					
No Fruit	Ref				
Fruit	-1.02[a]	-1.51	-0.53	**<0.001**	
Sexual Minoritized women					
No Fruit	Ref				
Fruit	-0.59[a]	-0.94	-0.24	**<0.001**	
**Product Use Intention†**					
E-liquid Bottle Shown					**0.046**
Heterosexual men					
No E-liquid Bottle	Ref				
E-liquid Bottle	-0.65	-0.90	-0.40	0.627	
Heterosexual women					

No E-liquid Bottle	Ref				
E-liquid Bottle	-0.77[a]	-1.22	-0.32	**0.001**	
Sexual Minoritized men					
No E-liquid Bottle	Ref				
E-liquid Bottle	-0.98[a]	-1.61	-0.35	**0.002**	
Sexual Minoritized women					
No E-liquid Bottle	Ref				
E-liquid Bottle	-0.91[a]	-1.30	-0.52	**<0.001**	
"Alternative to Cigarettes" Claim					**0.026**
Heterosexual men					
No Claim	Ref				
"Alternative to Cigarettes" Claim	-0.14	-0.57	0.29	0.541	
Heterosexual women					
No Claim	Ref				
"Alternative to Cigarettes" Claim	-0.46	-1.13	0.21	0.179	
Sexual Minoritized men					
No Claim	Ref				
"Alternative to Cigarettes" Claim	-0.46	-1.28	0.36	0.270	
Sexual Minoritized women					
No Claim	Ref				
"Alternative to Cigarettes" Claim	-0.78	-1.39	-0.17	**0.011**	

† Because their small sample size, transgender and nonbinary participants were excluded from multivariable analyses.

*P-values were calculated using Wald tests. Tukey's tests were used to assess statistical significance of pairwise comparisons. P-values that meet the criteria for statistical significance are bolded. Means with a common superscript letter differ (p<0.001). Superscript a denotes difference compared to heterosexual men, b denotes difference compared to heterosexual women, c denotes difference compared to sexual minoritized men, and d denotes difference compared to sexual minoritized women. Models analyzed the interaction between sexual orientation and gender identity and feature, but results are only stratified by sexual orientation/gender identity when the interaction was statistically significant. Stratified results are presented from models with statistically significant interactions between sexual orientation/gender identity and feature.

Supplemental Table 2: Participant Demographics (N=497) of 2021 Brief Online Advertisement Exposure Study, for subgroups: Heterosexual men, Heterosexual women, Gay men, Lesbian women, Bisexual men, Bisexual women, and Transgender/Nonbinary

	Hetero-sexual men (n=188)	Hetero-sexual women (n=82)	Gay men (n=27)	Lesbian women (n=20)	Bisexual men (n=23)	Bisexual women (n=122)	Transgender/ Nonbinary[b] (n=35)
Age; mean (sd)	31.7 (11.1)	36.9 (9.6)	33.8 (10.2)	27.2 (9.3)	31.9 (10.2)	26.7 (8.9)	26.1 (8.3)
Race/ethnicity; n(%)							
Non-Hispanic White	133 (70.7)	62 (75.6)	14 (51.9)	12 (60.0)	19 (82.6)	84 (68.9)	29 (82.9)
Non-Hispanic Black	31 (16.5)	6 (7.3)	9 (18.5)	2 (10.0)	1 (4.3)	11 (9.0)	0 (0.0)
Hispanic	10 (5.3)	5 (6.1)	3 (11.1)	1 (5.0)	1 (4.3)	9 (7.4)	4 (11.4)
Other/multiple	14 (7.4)	9 (11.0)	5 (18.5)	5 (0.25)	2 (8.7)	18 (14.8)	1 (2.9)
Income; n (%)							
<$50,000	50 (26.6)	57 (69.5)	15 (55.6)	12 (60.0)	10 (43.5)	97 (79.5)	29 (82.9)
≥$50,000	138 (73.4)	25 (30.5)	12 (44.5)	8 (40.0)	13 (56.5)	25 (20.5)	6 (17.1)
E-cigarette use[a]							
Never	53 (28.2)	35 (42.7)	11 (40.7)	9 (45.0)	10 (43.5)	40 (32.8)	9 (25.7)
Ever	26 (13.8)	30 (36.6)	6 (22.2)	3 (15.0)	4 (17.4)	37 (30.3)	16 (45.7)
Past 30-day	109 (58.0)	17 (20.7)	10 (37.0)	8 (40.0)	9 (39.1)	45 (36.9)	10 (28.6)
Cigarette use							
Never	35 (18.6)	31 (37.9)	7 (25.9)	10 (50.0)	5 (21.7)	58 (47.5)	12 (34.3)
Ever	44 (31.0)	31 (37.9)	14 (44.4)	7 (35.0)	12 (52.2)	37 (30.3)	19 (54.3)
Past 30-day	30 (21.1)	20 (24.4)	8 (29.6)	3 (15.0)	6 (26.1)	27 (22.1)	4 (11.4)
Alcohol use							
Never	8 (4.3)	4 (0.5)	0 (0.0)	2 (10.0)	3 (13.0)	6 (4.9)	1 (2.9)
Ever	12 (6.4)	14 (17.1)	6 (22.2)	5 (25.0)	2 (8.7)	22 (18.0)	13 (37.1)
Past 30-day	168 (89.4)	64 (78.0)	21 (77.8)	13 (65.0)	18 (70.3)	94 (77.0)	21 (60.2)
Mean (sd) of Perceived Advertise-ment	3.2 (1.2)	2.2 (1.1)	2.1 (1.1)	2.2 (1.1)	1.9 (1.0)	2.1 (1.0)	2.3 (1.1)

Effectiveness (1-5)							
Mean (sd) of Perceived Advertise-ment Relevance (1-5)	2.9 (1.4)	1.6 (0.9)	1.5 (0.9)	1.8 (1.0)	1.6 (0.9)	1.6 (0.9)	1.8 (1.1)
Mean (sd) of Product Use intention (1-5)	3.1 (1.5)	1.7 (1.0)	1.6 (0.9)	1.9 (1.2)	1.6 (1.1)	1.8 (1.1)	2.0 (1.2)

[a]Participants were asked to report their e-cigarette use and if they had ever “used an electronic cigarette (e-cigarette), even one or two times?” If participants answered “Yes,” they were then asked, “During the past 30 days, on how many days did you use an e-cigarette?” E-cigarette use status categorized as “current use” if they used an e-cigarette in the past 30 days, “ever use” if they ever used e-cigarettes but reported 0 days of e-cigarette use in the past 30 days, and “never use” if they responded “No” to ever using an e-cigarette even one or two times. Participants also reported their use of combustible cigarettes and alcohol. We recoded participants to “current,” “ever,” and “never” users of combustible cigarettes and alcohol similar to how we categorized e-cigarette use status.

© **2023 Liu J. et al.**
